# How Wastewater Addition Reshapes Peatland Vegetation via Linked Abiotic and Biotic Changes

**DOI:** 10.3390/biology14111611

**Published:** 2025-11-17

**Authors:** Yue Li, Xue Wang, Lianxi Sheng, Chunguang He, Haibo Jiang

**Affiliations:** 1 School of Geographical Sciences, Changchun Normal University, Changchun 130032, China; liy854@nenu.edu.cn; 2 Key Laboratory of Wetland Ecology and Vegetation Restoration, Ministry of Ecology and Environment, School of Environment, Northeast Normal University, Changchun 130117, China; shenglx@nenu.edu.cn (L.S.); he-cg@nenu.edu.cn (C.H.); jianghb625@nenu.edu.cn (H.J.)

**Keywords:** peatland, soil properties, plant nutrients, plant community structure, aboveground biomass

## Abstract

Peatlands, as globally important carbon sinks, are highly sensitive to human disturbances. The direct discharge or use of domestic sewage with different treatment gradients as peatland replenishment water affects plant communities, but how the plant community structure and biomass change and what drives these changes remain unclear. This study simulated domestic sewage input to a sedge-dominated peatland in the Changbai Mountain region of Northeast China, explored changes in plant community structure and aboveground biomass and revealed the key factors influencing these plant characteristics. The results revealed the following: (1) Reclaimed water treatment (Z) had little effect on the plant community structure; treatments with 50% tap water and 50% domestic sewage (H) and domestic sewage (W) caused rapid expansion of Poaceae plants, with *Echinochloa crus-galli* (L.) P. Beauv. occupying dominance. (2) Domestic sewage input increases plant diversity, which is driven jointly by soil nutrients and aboveground plant nutrients. (3) The H and W treatments significantly altered aboveground biomass, which was positively correlated with soil nutrients, aboveground plant nutrients, and diversity, whereas the Z treatment had little effect. This research provides scientific support for the control of domestic sewage discharge and whether reclaimed water can be used for ecological water replenishment in peatlands. It holds significant practical value for the scientific management of peatlands, the maintenance of carbon sink functions, and the mitigation of climate change.

## Simple Summary

This study aimed to investigate changes in peatland plant community structure, aboveground biomass, and key driving factors under simulated input conditions of reclaimed water, 50% tap water mixed with 50% domestic sewage (mixed water), and domestic sewage by conducting an experiment on sedge-dominated peatlands in the Changbai Mountains of Northeast China. The results indicated that the input of mixed water and domestic sewage promoted the rapid expansion of Poaceae plants. The input of domestic sewage at different treatment gradients increased plant diversity by influencing soil nutrients and aboveground plant nutrients. Moreover, soil nutrients, aboveground plant nutrients, and species diversity were the main factors driving the increase in aboveground biomass under the input of domestic sewage at different gradients. Additionally, reclaimed water input had little effect on the plant community structure and aboveground biomass. Therefore, the input of domestic sewage into peatlands alters the plant community structure by affecting abiotic and biological factors, promoting the transition of peatlands from Cyperaceae to Gramineae. This transition leads to an increase in aboveground plant biomass and increases the risk of peatlands shifting from a “carbon sink” to a “carbon source”. Reclaimed water holds great potential for water replenishment in peatlands.

## 1. Introduction

Although peatlands account for only 3% of the global land area, they store approximately one-third of the world’s soil carbon, making them a significant global carbon sink [1,2]. Due to the acidic, anaerobic, and low-temperature environment of northern peatlands, the decomposition of soil organic matter is slow. As a result, nutrient elements such as nitrogen and phosphorus exist mainly in organic forms, and there are insufficient available nutrients for plant absorption and utilization [3]. Consequently, oligotrophic plant communities have formed, making peatlands highly sensitive to external nutrient inputs [4,5]. With rapid economic development, the discharge of domestic sewage continues to increase. Domestic sewage primarily contains nutrients such as organic carbon, nitrogen, and phosphorus [6]. In many rural areas, domestic sewage is discharged directly into peatlands without treatment. Additionally, in some regions, domestic sewage with different treatment gradients is input into peatlands through water replenishment or other methods. These scenarios can increase soil nutrient availability, alter plant growth status and community structure, and ultimately affect ecosystem functions.

Plant communities are important components of peatlands, and their species composition and diversity are key drivers influencing the carbon sink function of peatlands [7]. As oligotrophic wetlands, northern peatlands promote plant growth and disrupt the competitive balance among species when limited nutrients are supplemented, thereby altering their community composition [8,9]. For example, a 14-year nitrogen addition experiment on peatlands revealed that the competitive advantages of the original dominant species *Sphagnum* and *Cladonia portentosa* decreased, with a significant reduction in coverage. In contrast, the growth of the graminoid plant *Eriophorum vaginatum* L. was promoted, and its coverage increased significantly, resulting in changes in the plant community structure [10]. At Mariana Lake Bog, vegetation changes occurred even under low nitrogen loads, and the peatland plant community showed progressive development during a 5-year nitrogen input study [11]. Researchers conducted a 9-year nitrogen and phosphorus input experiment on Mer Bleue Bog, which transformed the *Sphagnum* and dwarf shrub community into a taller shrub community without *Sphagnum* [12]. Furthermore, most studies have confirmed that long-term nutrient inputs can change the soil nutrient content, reduce soil nutrient heterogeneity, and thereby decrease plant diversity. Some studies have shown that plant diversity tends to decrease with increasing nitrogen input, and the abundance of plant species sensitive to nitrogen decreases significantly [13]. High-nitrogen inputs can cause soil acidification and nutrient imbalance, thereby affecting soil and plant health and leading to the loss of species diversity, especially in low-pH soil, as the species pool adapted to low pH is relatively small, resulting in reduced species richness [14,15]. However, there are also contradictory research conclusions. For example, nitrogen input increased the diversity of plant communities, mainly because it improved the nitrogen-deficient conditions of peatlands and promoted the growth of vascular plants [16]. Although studies on single-nutrient inputs have provided an important foundation for understanding peatland responses to environmental changes, they still have certain limitations. Currently, the primary human impact on peatlands is the input of multiple nutrients. Therefore, researching the effects of domestic sewage input with different treatment gradients (multiple nutrient synergistic inputs) on peatland plant community structure, as well as identifying the factors regulating plant diversity, can reveal the response mechanisms of peatland ecosystems under multifactor interactions. This provides more targeted strategies for protecting peatland plant communities and their ecological functions and holds significant practical significance.

Biomass is a direct indicator of plant growth status and ecosystem productivity and plays a crucial role in maintaining the stability of plant communities and the balance of ecosystem functions [17]. In oligotrophic wetlands, nutrients are usually the key factors limiting plant biomass accumulation [18]. The input of external nutrients alleviates soil poverty, and an increase in soil nutrients can promote plant growth, thereby increasing plant biomass. Studies have shown that, among soil properties, the soil organic carbon (SOC) content significantly affects biomass. With increasing SOC content, nutrient availability improves, which is more conducive to the growth of species with high growth rates and dense root systems, ultimately increasing biomass [19]. Other studies have reported that external nitrogen input increases the content of available nitrogen in the soil, thereby promoting the accumulation of plant biomass [13]. In addition, plant biomass is closely related to plant community competition and functional traits. The input of external nutrients can improve the nutrient absorption efficiency of plants, increase the accumulation of plant nutrients, and significantly promote the growth of nutrient-demanding plants, leading to an increase in plant biomass [20]. For example, with increasing nitrogen input, the main nitrogen absorber in the peatland ecosystem gradually shifts from *Sphagnum* to shrubs. The competitive advantage of *Sphagnum* declines, and the nitrogen content in the leaves of shrubs such as *Andromeda polifolia* L. increases. This accelerates shrub growth and significantly increases shrub coverage, resulting in an increase in plant biomass [11]. Moreover, some studies have shown that plant diversity has a significant effect on aboveground biomass, with greater species diversity in plant communities leading to greater aboveground biomass [21,22]. Therefore, clarifying the variation characteristics of aboveground plant biomass in peatlands under external nutrient inputs and their influencing factors is crucial for predicting the response of peatland ecosystem functions to environmental changes. However, the variation characteristics of aboveground plant biomass in peatlands and their biotic and abiotic influencing factors under the input of domestic sewage with different treatment gradients remain unclear.

Changbai Mountain is a major distribution area of northern peatlands in China, as well as an important ecological barrier and water source in Northeast China [23]. However, problems such as the direct discharge of domestic sewage into peatlands and the high cost of long-distance water transfer for the ecological water replenishment of peatlands are prominent. Therefore, our study took a sedge-dominated peatland in the Changbai Mountain region as the research object, using domestic sewage, 50% tap water mixed with 50% domestic sewage, and reclaimed water as input water sources. A simulated control experiment was conducted to analyze the changes in the peatland plant community structure, aboveground plant biomass, soil properties, and nutrient accumulation in aboveground plant parts. The objectives of this study were to (1) clarify how the species composition, diversity of plant communities, and aboveground plant biomass change after the input of domestic sewage with different treatment gradients into sedge-dominated peatlands; (2) identify the key factors regulating the changes in plant diversity and aboveground biomass; (3) determine whether reclaimed water can replace long-distance water transfer for peatland water replenishment. Based on the above, we hypothesize that: (1) the input of domestic sewage with different treatment gradients into sedge-dominated peatlands increases plant diversity and biomass, accompanied by the expansion of Poaceae plants and a decrease in the dominance of sedges; (2) the input of domestic sewage with different treatment gradients enhances plant diversity by affecting soil nutrient contents and aboveground plant nutrient contents; (3) changes in soil nutrients, aboveground plant nutrients, and plant diversity jointly drive the increase in aboveground plant biomass; (4) reclaimed water had little effect on the plant community. The research results can provide a scientific basis for the ecological protection and scientific management of peatlands in the Changbai Mountain region of Northeast China.

## 2. Materials and Methods

### 2.1. Experimental Materials

**Figure 1:**
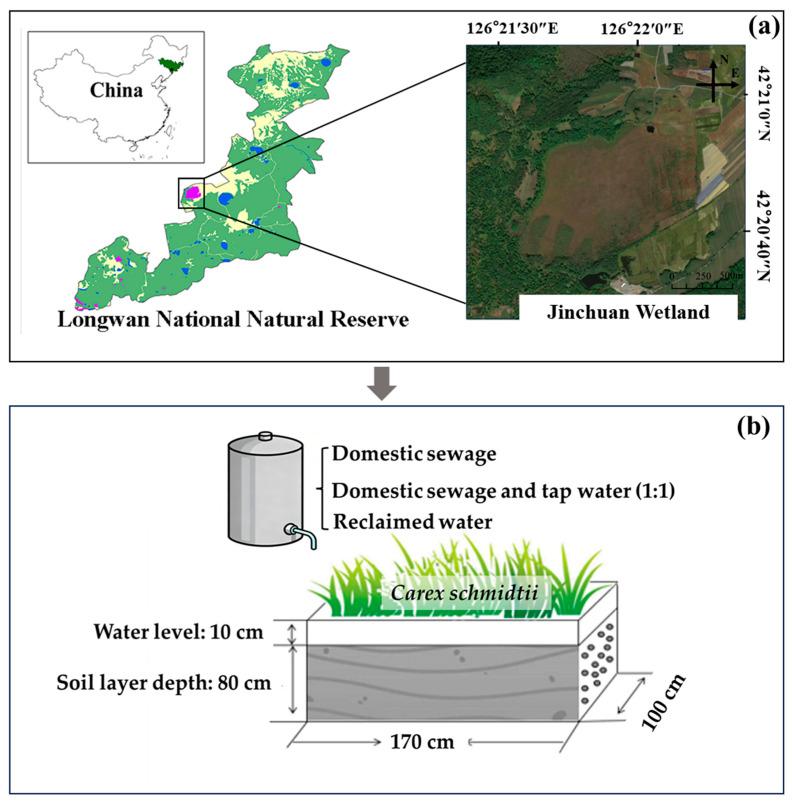
Sampling point location and simulation experiment layout: (**a**) simulation experiment sampling point location; (**b**) schematic diagram of the simulation experiment.

This study adopted a simulation experiment. The soil and plants used in the study were collected from the Jinchuan Wetland, a typical peatland in Jinchuan town, Jilin Province (42°20′56″ N, 126°22′51″ E; Figure 1a). The annual temperature range in this area is from −40 °C to 30 °C, with an average annual temperature of 3.3 °C. The average annual rainfall in the past 40 years has ranged from 600 to 1100 mm, and the frost-free period is approximately 135 days. It covers an area of approximately 73.1 hm^2^ and belongs to a small basin type and ombrotrophic peatland. The peat layer has a clear vertical profile structure, showing regular changes from top to bottom: the surface layer (0–30 cm) is a brownish-black to black, loose and porous organic layer; the middle layer (30–60 cm) is dark brown to black, relatively dense in texture with many residual plant fibers; and the bottom layer (60–80 cm) is black, compact in texture, with highly decomposed plant residues and a transition to humified peat. The plant community structure is relatively uniform, with *Carex schmidtii* Meinsh. as the dominant species. The simulation experiment was carried out at the Longwan Wetland Ecological Experimental Station in Jinchuan town, Jilin Province. In late June 2020, peat soil was collected from the Jinchuan Wetland, transported to the experimental station, and homogenized. It was then transferred into the experimental devices to ensure consistency in their initial conditions (Figure 1b). The dimensions of the experimental device in this study are 1.7 × 1 × 1 m. This specification refers to the design of controlled experiments in fields such as constructed wetlands and peatland ecological effects, enabling effective simulation of the characteristics of small-scale peatland microhabitats. With a depth of 1 m, the device can fully cover the soil profile of peatlands. Moreover, its spatial capacity is sufficient to ensure the natural growth density and structural integrity of the plant community, thus guaranteeing the reliability of community-level data [24]. The filling depth of the peat soil was 80 cm. After filling, the tap water was irrigated, and the water level was maintained at approximately 10 cm, which was the same as the water level at the soil sampling site in the Jinchuan wetland.

In August 2020, plant transplantation was initiated. *Carex schmidtii* with uniform growth and a height of approximately 30 cm were selected, and the planting density was consistent with that of *Carex schmidtii* in the Jinchuan wetland. The growth of *Carex schmidtii* in each device was subsequently observed, and the experiment officially began in May 2021 after *Carex schmidtii* grew stably. Before the start of the experiment (on 15 April 2021), soil and water samples were collected to determine the background values. The soil properties are shown in Table 1, and the concentration data of various water quality indicators are presented in Table 2. Domestic sewage was collected from the domestic sewage septic tank of the experimental station. The concentrations of various indicators in reclaimed water were formulated in accordance with the first-level standards specified in the “Discharge Standard of Pollutants for Municipal Wastewater Treatment Plants of China”. Tap water was used for the control treatment. The concentrations of water quality indicators in the experimental water are shown in Table 3. The meteorological data collected during the experiment are presented in Tables S1 and S2.

### 2.2. Experimental Design

The experiment was conducted from May to September 2021 and 2022, covering two growing seasons. The experimental water flowed controllably into the experimental device via gravity from a water tank (with a volume of 450 L) located approximately 10 cm above the experimental device. Domestic sewage collected from the septic tank of the experimental station was uniformly mixed and then fed into the experimental device to ensure consistent influent concentrations among the three parallel treatment groups. The water filling volume in each experimental device was kept consistent. The water level in this simulation experiment was maintained at approximately 10 cm, which is a suitable water level for the growth of *Carex schmidtii* [25]. When the water level dropped below 10 cm, the water was replenished immediately, and the amount of water replenished each time was the same for all the treatments. In addition, a movable rain shelter was installed above the experimental devices to prevent rainfall from entering the devices and affecting the experimental results.

A total of 4 treatments were used in this experiment, namely, the control treatment (tap water, CK), Z treatment, H treatment, and W treatment, with 3 replicates for each treatment.

### 2.3. Soil Sample Collection and Determination

On 17 July and 4 September 2022, soil samples from the 0–20 cm depth were collected. Five sampling points were selected via the “S” sampling method, and 5 peat columns were drilled via a peat auger. The sample size was 24. SOC was determined via the potassium dichromate oxidation external heating method [26]; TN was determined via the Kjeldahl method [27]; ammonium nitrogen (NH_4_^+^-N) and nitrate nitrogen (NO_3_^−^-N) were extracted via the KCl extraction method [28], and the extract was determined via a fully automatic chemical analyzer (Smart Chem 200, AMS Westco, Italy); TP was digested via the HClO_4_-H_2_SO_4_ method and determined via the molybdenum antimony anti-colorimetric method [29]; available phosphorus (AP) was determined via the hydrochloric acid sulfuric acid extraction method [30]; pH was determined via the potentiometric method; and EC was determined via the electrode method.

### 2.4. Determination Indicators and Methods for Plants

#### 2.4.1. Nutrients and Biomass in Aboveground Plant Parts

On 17 July and 4 September 2022, a 0.5 × 0.5 m quadrat was placed in the experimental area for plant sample collection, with a sample size of 24.

In this simulated control experiment, the soil was thoroughly homogenized before being filled into the experimental devices, and the transplanted plants exhibited consistent growth vigor. Therefore, the spatial heterogeneity in the experimental area was relatively low. Additionally, the quadrat method was adopted for multipoint sampling, and the collected samples were fully mixed prior to determination to ensure the statistical representativeness of the plant nutrient data. The collected aboveground parts of the plants were dried and ground for the determination of nutrient indicators. The aboveground parts of the collected plants were dried and ground for the determination of nutrient indicators. The plant aboveground carbon (TC) content was determined via the potassium dichromate sulfuric acid oxidation method [26], the plant aboveground nitrogen (TN) content was determined via the Kjeldahl method [31], and plant aboveground phosphorus (TP) content was determined via the molybdenum antimony anti-colorimetric method [32]. The aboveground biomass was obtained by harvesting the aboveground parts of the plants and weighing the dry weight on 4 September, with a sample size of 12.

#### 2.4.2. Plant Diversity

On 4 September 2022, the number of species in the 0.5 × 0.5 m quadrat was counted to calculate plant diversity. Plant diversity was expressed via the Richness index and Shannon–Wiener index, and the calculation formulas were as follows [33]:

Richness index:

(1) R = S

Shannon–Wiener index:

(2) $$H\;=-\sum_{i=1}^{S}P_{i}\mathrm{Ln}(P_{i})$$

where *S* represents the number of species in the plant community and P_i_ is the proportion of the ith species in the total number of species.

### 2.5. Statistical Analysis

The standard deviation of the data was calculated via STDEVP in Excel. Before statistical analysis, all the data were subjected to the Shapiro–Wilk test and Levene’s test. One–way analysis of variance and Tukey’s post hoc analysis were used to test the differences in soil parameters (SOC, TN, NH_4_^+^-N, NO_3_^−^-N, TP, AP, pH, and EC) and plant traits (nutrient content in aboveground plants, species richness, Shannon index, and aboveground biomass) among the different treatments. Pearson correlation analysis was used to explore the correlations between soil parameters (SOC, TN, NH_4_^+^-N, NO_3_^−^-N, TP, AP, pH, and EC), plant traits (nutrient content in aboveground plants, species richness, and the Shannon–Wiener index) and aboveground plant biomass (SPSS 22.0). Boruta was used to identify the key factors influencing plant diversity (Boruta’ R package, version 4.0.2). First, the shadow features were constructed, and both the original features and the shadow features were input into the model. The importance of all the features is calculated. Then, the irrelevant features are gradually eliminated, and the above process is repeated until all the features have completed classification or until the set number of iterations is reached. Finally, the important features are output [34].

## 3. Results

### 3.1. Soil Properties

**Figure 2:**
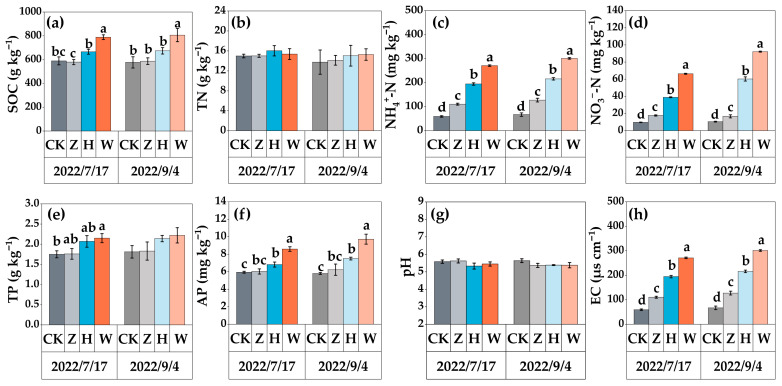
Soil physical and chemical properties under different experimental treatments: (**a**) SOC; (**b**) TN; (**c**) NH_4_^+^-N; (**d**) NO_3_^−^-N; (**e**) TP; (**f**) AP; (**g**) pH; (**h**) EC. The error bars represent the standard deviation among three replicates. Different lowercase letters indicate significant differences between treatment groups (*p* < 0.05).

After two growing seasons of the experiment, changes in the soil properties were observed (Figure 2). Compared with the CK treatment, the Z treatment did not significantly affect the contents of SOC, TN, TP, and AP or pH but significantly increased the contents of soil NH_4_^+^-N, NO_3_^−^-N, and EC (*p* < 0.01). The H and W treatments increased the SOC and TP contents, had no significant effect on the soil pH, and significantly increased the contents of NH_4_^+^-N, NO_3_^−^-N, AP, and EC (*p* < 0.01). Compared with those in the CK treatment group, the NH_4_^+^-N content in the H and W treatment groups increased by 68.99% and 77.77%, the NO_3_^−^-N content increased by 82.60% and 88.62%, and the AP content increased by 22.67% and 40.33%, respectively.

### 3.2. Plant Nutrient Dynamics

**Figure 3:**
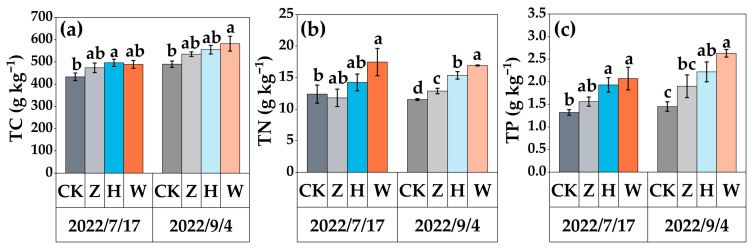
Distribution characteristics of nutrients in aboveground plant parts under different experimental treatments: (**a**) TC content in aboveground plant parts; (**b**) TN content in aboveground plant parts; (**c**) TP content in aboveground plant parts. The error bars represent the standard deviation among three replicates. Different lowercase letters indicate significant differences between treatment groups (*p* < 0.05).

The inputs of reclaimed water, 50% tap water mixed with 50% domestic sewage, and domestic sewage into the peatland increased nutrient accumulation in the aboveground parts of the plants (Figure 3). Especially at the end of the second year of the experiment, the TC content in the aboveground parts of the plants in the Z treatment increased to 534.81 g·kg^−1^, and the TN and TP contents increased to 12.87 g·kg^−1^ and 1.9 g·kg^−1^, respectively. Compared with those in the CK treatment, the contents of TC, TN, and TP in the aboveground parts of the plants in the H and W treatments increased. The TC content of the plants increased by 11.75% and 15.83%, the TN content increased by 25.0% and 31.96%, and the TP content increased by 34.68% and 44.87%, respectively.

### 3.3. Changes in Plant Community Composition and Diversity Indices

**Figure 4:**
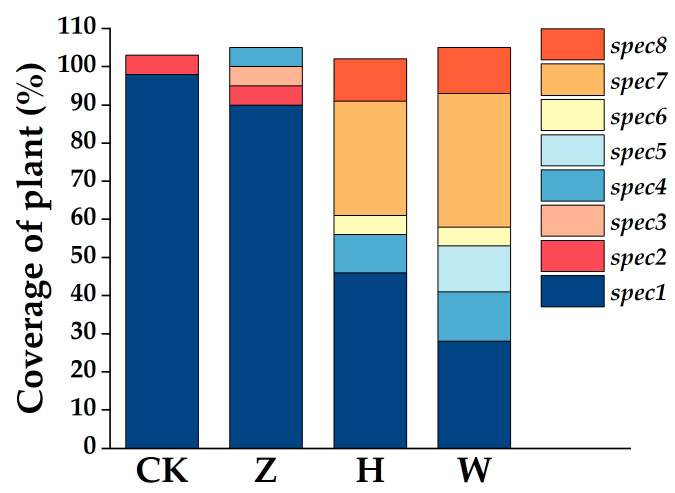
Plant community composition and species coverage under different experimental treatments. spec1. *Carex schmidtii* Meinsh.; spec2. *Thelypteris palustris* (L.) Schott; spec3. *Stachys japonica* Miq.; spec4. *Calla palustris* L.; spec5. *Scirpus validus* Vahl.; spec6. *Sagittaria trifolia* subsp. Leucopetala; spec7. *Echinochloa crus-galli* (L.) P. Beauv.; spec8. *Digitaria sanguinalis* (L.) Scop.

**Figure 5:**
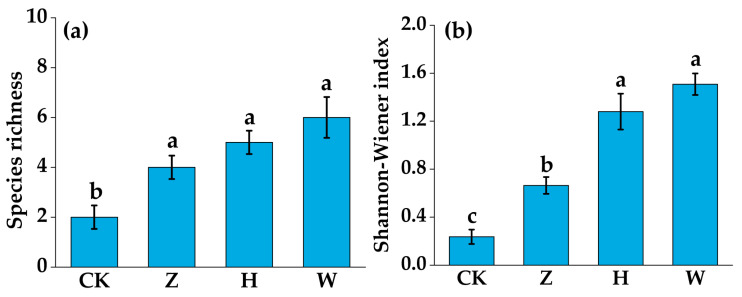
Plant community diversity under different experimental treatments: (**a**) Richness index; (**b**) Shannon–Wiener index; error bars represent the standard deviation among three replicates. Different lowercase letters indicate significant differences between treatment groups (*p* < 0.05).

The survey results from the second year of the experiment revealed that the plant community in the CK treatment mainly included *Carex schmidtii* and *Thelypteris palustris* (L.) Schott, with coverage rates of 98% and 5%, respectively. The plant community in the Z treatment mainly consisted of *Carex schmidtii*, *Stachys japonica* Miq., *Thelypteris palustris*, and *Calla palustris* L., with *Carex schmidtii* coverage of 90%. The plant community in the H treatment mainly included *Carex schmidtii*, *Echinochloa crus-galli*, *Digitaria sanguinalis* (L.) Scop, *Calla palustris*, and *Sagittaria trifolia* subsp. Leucopetala, among which the coverage of *Carex schmidtii* decreased to 46% and that of *Echinochloa crus-galli* decreased to 30%. In the plant community of the W treatment, the coverage of *Carex schmidtii* was only 28%, and other species included *Calla palustris*, *Scirpus validus*, *Sagittaria trifolia* subsp., *Echinochloa crus-galli* and *Digitaria sanguinalis*, with the coverage of Poaceae plants accounting for more than 40% (Figure 4). After only two years of the experiment, the plant community structure changed significantly with the addition of 50% tap water mixed with 50% domestic sewage and domestic sewage. The coverage of the original dominant species *Carex schmidtii* was only 46% and 28%, and the coverage of *Echinochloa crus-galli* in the domestic sewage treatment group reached 35%, replacing *Carex schmidtii* as the dominant species. The plant species richness of the CK, Z, H, and W treatment groups was 2, 4, 5, and 6, respectively, and the Shannon–Wiener indices were 0.24, 0.66, 1.28, and 1.51, respectively. The inputs of reclaimed water, 50% tap water mixed with 50% domestic sewage, and domestic sewage into the peatland increased plant diversity (*p* < 0.01, Figure 5). Compared with that in the CK treatment, the plant community structure in the H and W treatment groups changed significantly.

### 3.4. Variation Characteristics of Aboveground Plant Biomass

**Figure 6:**
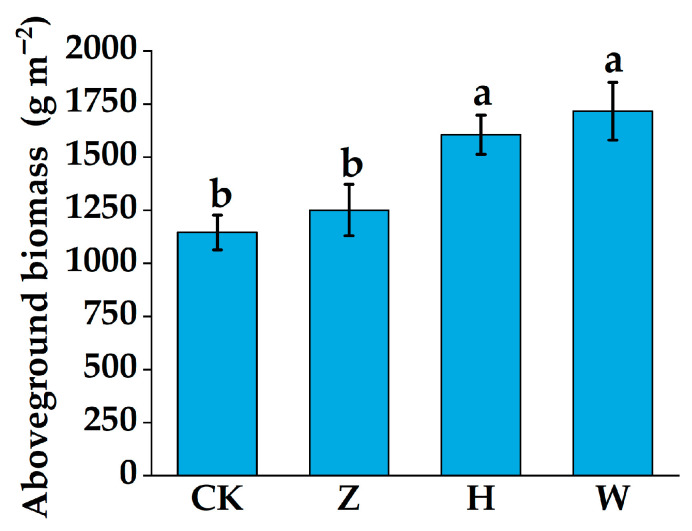
Aboveground plant biomass under different experimental treatments. The error bars represent the standard deviation among three replicates. Different lowercase letters indicate significant differences between treatment groups (*p* < 0.05).

The input of domestic sewage at different treatment gradients increased the aboveground biomass of plants. Compared with those in the CK treatment group (1145.33 g·m^−2^), the aboveground biomass of the plants in the Z, H, and W treatment groups increased by 8.42%, 28.65%, and 33.27%, respectively. The aboveground plant biomass of the H and W treatment groups was significantly greater than that of the CK treatment group (*p* < 0.01, Figure 6).

### 3.5. Effects of Soil Parameters and Plant Traits on Plant Diversity and Aboveground Biomass

**Figure 7:**
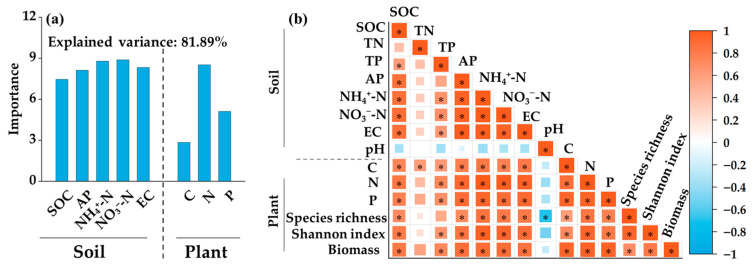
Main factors affecting plant community diversity and aboveground biomass: (**a**) Results of Boruta feature selection; (**b**) Pearson correlation analysis. * indicates a significant correlation at the 0.05 level.

Boruta was used to identify the important variables affecting the species diversity index (Figure 7a). The soil SOC, AP, NH_4_^+^-N, NO_3_^−^-N, and EC and plant TC, TN, and TP all had significant effects on the species diversity index, and the importance of the soil NH_4_^+^-N, NO_3_^−^-N, and TN contents in the aboveground plant parts was relatively high. The results of the Pearson correlation analysis revealed that the aboveground plant biomass was significantly positively correlated with the soil SOC, TP, AP, NH_4_^+^-N, NO_3_^−^-N, and EC, and the contents of TC, TN, and TP in the aboveground plants, species richness, and the Shannon–Wiener index had a significant positive impact on the aboveground plant biomass (*p* < 0.05, Figure 7b).

## 4. Discussion

### 4.1. Effects of Domestic Sewage with Different Treatment Gradients on Plant Community Structure

The input of domestic sewage with different treatment gradients during two consecutive growing seasons changed the plant community composition of the peatland. In particular, the plant community structure of the treatments with 50% tap water mixed with 50% domestic sewage and pure domestic sewage changed significantly. The coverage of *Carex schmidtii* decreased to 46% and 28%, respectively, and the Poaceae plant *Echinochloa crus-galli* in the domestic sewage treatment group replaced *Carex schmidtii* as the dominant species (Figure 4). The above research results are attributed mainly to the combined effects of the activation of the existing gramineous seed bank in the soil and the differences in species competitive advantages under nutrient enrichment. The peatland dominated by *Carex schmidtii* retains a dormant seed bank of gramineous plants such as *Echinochloa crus-galli*. Under oligotrophic conditions, these seeds cannot germinate or grow due to nutrient limitations. After the application of domestic sewage to the peatland, the nutrients such as organic matter, nitrogen, and phosphorus contained in the sewage continuously accumulate in the soil. This effectively alleviates the nutrient limitation of the peat soil, prevents nutrient restriction for seed germination, and activates the stored seed bank of gramineous plants such as *Echinochloa crus-galli* in the soil [35]. In addition, as a species with high nutrient requirements, *Echinochloa crus-galli* exhibit relatively strong growth and competitive advantages in nutrient-enriched environments. It has a high growth rate and strong tillering ability, leading to rapid expansion and a significant increase in coverage, thereby dominating the plant community of the peatland [36]. As an indicator plant of nutrient-limited peatlands, *Carex schmidtii* has a low utilization efficiency of nutrients such as nitrogen and phosphorus [37]. With increasing soil nutrient content, their competitive advantage decreased, and their coverage significantly decreased.

In addition, this study revealed that the input of domestic sewage with different treatment gradients significantly increased plant diversity in peatlands (Figure 5). This is related mainly to the increase in soil nutrient content (Figure 7a), especially soil NH_4_^+^-N and NO_3_^−^-N. As the forms of available nitrogen can be directly absorbed by plants, increasing their content is the key to relieving the growth limitations of Poaceae plants [38]. Compared with sedge plants, Poaceae plants have greater nitrogen use efficiency and greater competitiveness for underground nutrients [39]. Therefore, when the input of domestic sewage with different treatment gradients into the peatland significantly increased the contents of soil NH_4_^+^-N and NO_3_^−^-N, the rapid absorption capacity of the roots of *Echinochloa crus-galli* for available nitrogen was transformed into a growth advantage, resulting in significant expansion. Moreover, input of domestic sewage reduced the soil N/P ratio, which indicated the priority alleviation of phosphorus limitation existing in oligotrophic peatlands. As a key element for plant nucleic acid synthesis and energy metabolism, a sufficient phosphorus supply broke the original nutrient limitation pattern, creating conditions for the colonization of associated species such as *Stachys japonica* and *Calla palustris*, thus leading to a significant increase in plant species [40]. Additionally, the increase in SOC content not only enhanced soil water and nutrient retention capacities but also extended the nutrient supply period, further optimizing the soil microenvironment [41]. It synergistically promoted the growth of eutrophic plants with nitrogen and phosphorus elements, thereby further improving plant diversity. The Boruta results of this study revealed that the contents of TC, TN, and TP in plants also had a significant effect on species diversity (Figure 7a), which reflects that the ability of plants to absorb and utilize nutrients is a key factor affecting diversity. The input of domestic sewage with different treatment gradients into peatlands introduces nutrients such as organic carbon, nitrogen, and phosphorus, leading to an increase in the accumulation of carbon, nitrogen, and phosphorus nutrients in the aboveground parts of plants with high nutrient requirements, and their competitiveness and adaptability also improve accordingly [42]. This growth advantage allows plants such as *Echinochloa crus-galli*, *Digitaria sanguinalis*, *Scirpus validus*, and *Stachys japonica* to consume more resources and space in the community, thereby increasing plant diversity. In addition, nitrogen is the primary nutrient factor limiting plant growth. The increase in nitrogen content in the aboveground parts of plants not only indicates that there are sufficient nutrients available for plants such as *Echinochloa crus-galli* and *Digitaria sanguinalis* but can also significantly increase their photosynthetic efficiency, providing more energy for growth and reproduction [43], thereby changing the competitive pattern of the community, strengthening niche differentiation among species, and ultimately improving the plant diversity of the peatland.

### 4.2. Effects of Domestic Sewage with Different Treatment Gradients on Aboveground Plant Biomass

This study revealed that the input of domestic sewage with different treatment gradients promoted the aboveground plant biomass of the peatland (Figure 6). Consistent with previous research results, the increase in aboveground biomass was caused mainly by the increase in the biomass of Poaceae plants [44]. In this study, two Poaceae plants, *Echinochloa crus-galli* and *Digitaria sanguinalis*, made particularly significant contributions to the increase in biomass. The results of the Pearson correlation analysis revealed that the aboveground plant biomass was significantly positively correlated with the contents of nutrients such as soil NH_4_^+^-N, NO_3_^−^-N, and AP, as well as nutrient accumulation in the aboveground parts of the plants (Figure 7b). The input of external nutrients may affect aboveground plant biomass by influencing the habitat resource conditions on which plants depend or by directly affecting plant growth [45]. In this study, the input of domestic sewage increased the content of nutrients in the peatland soil, alleviating the nitrogen and phosphorus limitations of the ecosystem. It also enhanced microbial activity, accelerated the decomposition and transformation of soil nutrients, and provided a continuous nutrient supply for plants [46]. Moreover, domestic sewage input also regulated the activities of soil enzymes such as urease and phosphatase, promoting the release of available nutrients, including ammonium nitrogen and available phosphorus [47]. The roots of *Echinochloa crus-galli* and *Digitaria sanguinalis* are relatively developed, and they can respond quickly to increases in the contents of nutrients such as NH_4_^+^-N and NO_3_^−^-N, with high nutrient use efficiency, which enables them to grow rapidly and significantly increase their coverage and biomass [48]. Although the coverage and biomass of *Carex schmidtii* decreased accordingly, the high contribution rates of *Echinochloa crus-galli* and *Digitaria sanguinalis* to biomass ultimately led to an increase in aboveground plant biomass. Moreover, the input of domestic sewage into the peatland provided a nutrient-rich growth environment for Poaceae plants, and they allocated more resources to the aboveground parts to obtain sufficient light resources, resulting in an increase in the synthesis of photosynthetic products, thereby increasing the aboveground biomass [49].

Previous studies have shown that the maintenance of productivity in ecosystems largely depends on species diversity. The greater the species diversity of a plant community is, the greater its aboveground biomass is [22,50]. Our research reached the same conclusion that species richness and the Shannon–Wiener index had a significant positive effect on the aboveground plant biomass (Figure 7b), indicating that the input of domestic sewage with different treatment gradients into the peatland can have a positive effect on the aboveground plant biomass by increasing plant diversity. The ‘selection effect’ can explain the main mechanism by which species diversity affects aboveground plant biomass in this study. The ‘selection effect’ is very important when one or a few species contribute disproportionately to community value [51], and it mainly refers to a specific effect in which an increase in the number of species in a plant community increases the probability of selecting high-yield species from the species pool, thereby leading to an increase in plant biomass [52]. In this study, the input of domestic sewage into the peatland increased the coverage of high-productivity plants such as *Echinochloa crus-galli*, which greatly contributed to plant biomass. *Echinochloa crus-galli* can dominate the resource utilization of light, water, and nutrients in the community, thereby directly increasing the aboveground biomass of the entire plant community. Notably, the increase in plant diversity also activates microbial activity and accelerates organic carbon mineralization [53]. In addition, the increase in plant aboveground biomass leads to more oxygen consumption during litter decomposition, promoting the production of more methane by anaerobic microorganisms, which is not conducive to carbon accumulation [54]. Therefore, the input of domestic sewage may reduce the carbon sink function of peatlands.

In addition, some studies have reported that with increasing soil EC, plant diversity tends to decrease [55]; the input of domestic sewage leads to the accumulation of salts in the soil, and excessive salt affects the absorption of nutrients by plant roots, inhibits plant growth, and reduces plant biomass [56]. However, in this study, with increasing soil EC, plant diversity and biomass tended to increase. This may be because the experiment lasted only two growing seasons, and the salts in the domestic sewage may have only exerted a certain effect on the tissues and physiological processes within the plants without affecting plant growth.

This study revealed the ecological responses of peatland plant communities to different gradients of domestic sewage input, providing a scientific basis for the ecological restoration and pollution control of peatlands affected by wastewater and providing a reference for the scientific management of peatlands. It is of great value in maintaining the carbon sink function of global peatlands, mitigating climate change, and protecting biodiversity. This study also provides basic data support for cross-border wetland ecological protection cooperation. However, this study has certain limitations. First, the experiment only lasted for two growing seasons, which is a relatively short period. The ecosystem response is lagging, and the cumulative effects of salts, organic matter, and nutrients in domestic sewage may trigger more complex long-term ecological changes, such as plant community succession, which are difficult to fully manifest in a short time. Therefore, the temporal stability of the results of this study still needs to be further verified. Second, this study only measured the above-ground biomass of plants and lacked data on underground biomass. The input of domestic sewage may affect root growth and biomass allocation, thereby changing the stability and carbon sink function of the peatland ecosystem. Third, the frequency of peatland water replenishment has no fixed pattern. However, in this experimental mode, “replenishing water when the water level drops below 10 cm” ensures that the replenishment frequency is relatively stable. This approach fails to restore the randomness and intermittency of water replenishment in peatlands. Our research maintained the water level at approximately 10 cm, which ensured the controllability of the experiment but did not simulate the natural fluctuation characteristics of the water level in peatlands and ignored the potential impact of water level fluctuations on the peatland ecosystem. The experimental setup has limited space, which may restrict the development of plant root systems and the lateral diffusion of nutrients. Moreover, mesocosm dimensions can simulate only the local microenvironment and cannot cover the spatial heterogeneity of wild peatlands. Fourth, this study only conducted sampling during the growth period and withering period of the plants. There is no information on the number of repeated experiments in terms of the time dimension, which may limit the identification of the dynamic change patterns of the plant community. Therefore, in the future, large-scale and long-term continuous monitoring experiments will be carried out, and the sampling frequency will be increased to compensate for the aforementioned deficiencies. Furthermore, since this experiment is a short-term observation, if the input of domestic sewage is stopped in the future, the nutrient enrichment effect of the peatland will gradually weaken, thereby inhibiting the excessive expansion of grass species. The adaptive advantage of the original dominant species, *Carex schmidtii*, may become apparent, thereby promoting the gradual recovery of the plant community. The long-term recovery effect mentioned above is another direction we will focus on in the future. In the future, we will further clarify its recovery rate, stable state and key influencing factors by extending the observation period and conducting targeted restoration experiments to provide a more reliable scientific basis for the ecological restoration of peatlands.

## 5. Conclusions

Through the two-year simulation experiment, it was concluded that the input of domestic sewage with different treatment gradients into the sedge-dominated peatland changed the plant community composition and diversity, mainly by increasing the soil nutrient content and plant nutrient accumulation. The increase in the contents of nutrients such as soil NH_4_^+^-N and NO_3_^−^-N and the improvement in the accumulation of carbon, nitrogen, and phosphorus nutrients in plants promote the growth of plants such as *Echinochloa crus-galli*, *Digitaria sanguinalis*, and *Scirpus validus*, which have high nutrient requirements, and improve plant diversity. With the continuous input of domestic sewage, the increase in the number and coverage of high-productivity Poaceae species, such as *Echinochloa crus-galli*, increases the aboveground biomass of the plant community. In conclusion, biotic and abiotic factors jointly affect the plant diversity and aboveground biomass of peatlands, driving the transformation of peatlands from Cyperaceae to Gramineae under the conditions of 50% tap water mixed with 50% domestic sewage and domestic sewage. This vegetation type transformation triggers broader ecological effects: Poaceae plants have a higher litter decomposition rate than sedges, which reduces the long-term carbon sequestration efficiency of peat and slows peat accumulation; moreover, the change in vegetation type reshapes the rhizosphere microenvironment, potentially enhancing methanogen activity and thereby increasing methane emissions, ultimately increasing the potential risk of peatlands shifting from carbon sinks to carbon sources. Therefore, enhancing the treatment and pollution control of domestic sewage is necessary to reduce its potential negative impact on the structure and function of the peatland ecosystem. Reclaimed water has little effect on the plant community structure and aboveground biomass of peatlands. If appropriate input methods are adopted (alternating inputs of clean water and reclaimed water or pulse inputs of reclaimed water when the peatland is short of water) or if the reclaimed water is diluted and then input into the peatland, the reclaimed water may be a sustainable, environmentally friendly, and resource-saving water source for replenishing the peatland.

## Figures and Tables

**Table 1:** Soil physical and chemical properties.

SOC (g kg^−1^)	TN (g kg^−1^)	TP (g kg^−1^)	pH	EC (μs cm^−1^)
520.56 ± 20.43	15.41 ± 1.85	1.31 ± 0.29	5.83 ± 0.02	62.6 ± 1.63

Notes: Total nitrogen (TN), total phosphorus (TP), conductivity (EC).

**Table 2:** Concentration of the water quality index in peatland before the experiment.

COD (mg L^−1^)	TN (mg L^−1^)	NH_4_^+^-N (mg L^−1^)	TP (mg L^−1^)
3.75 ± 0.56	0.76 ± 0.03	0.38 ± 0.03	0.06 ± 0.01

Notes: Chemical oxygen demand (COD), total nitrogen (TN), ammonium nitrogen (NH_4_^+^-N), total phosphorus (TP).

**Table 3:** Properties of the experimental water.

	COD (mg L^−1^)	TN (mg L^−1^)	NH_4_^+^-N (mg L^−1^)	TP (mg L^−1^)
Domestic sewage	≤350	≤40	≤30	≤5
Domestic sewage and tap water (1:1)	≤175	≤20	≤15	≤2.5
Reclaimed water	≤50	≤15	≤5	≤0.5

## Data Availability

All data generated or analyzed during this study are included in the article.

## Supplementary Materials

The following supporting information can be downloaded at: https://www.mdpi.com/article/10.3390/biology14111611/s1, Table S1: Daily Temperature Chart (May-September 2021); Table S2: Daily Temperature Chart (May-September 2022); Table S3: Plant traits under different experimental treatment conditions; Table S4: Soil properties under different experimental treatment conditions.
