# Supplementary material for: How Wastewater Addition Reshapes Peatland Vegetation via Linked Abiotic and Biotic Changes
Source: Biology (Basel). 2025 Nov 17;14(11):1611. doi: 10.3390/biology14111611 (PMC12650231; doi:10.3390/biology14111611)
Supplement: Supplementary file 1 [file biology-14-01611-s001.zip › biology-3959732-supplementary.pdf]

Table S1. Daily Temperature Chart (May-September 2021).

	May		June		July		August		September	
	Daily Maximum Temperature	Daily Minimum Temperature	Daily Maximum Temperature	Daily Minimum Temperature	Daily Maximum Temperature	Daily Minimum Temperature	Daily Maximum Temperature	Daily Minimum Temperature	Daily Maximum Temperature	Daily Minimum Temperature
1	14°C	0°C	23°C	13°C	27°C	18°C	30°C	21°C	26°C	12°C
2	17°C	4°C	21°C	12°C	29°C	17°C	25°C	19°C	26°C	12°C
3	21°C	7°C	19°C	10°C	25°C	16°C	29°C	20°C	25°C	10°C
4	9°C	2°C	20°C	11°C	25°C	16°C	29°C	22°C	25°C	13°C
5	18°C	8°C	17°C	10°C	25°C	18°C	29°C	20°C	27°C	14°C
6	22°C	10°C	21°C	10°C	27°C	20°C	27°C	18°C	25°C	15°C
7	14°C	6°C	25°C	10°C	29°C	19°C	28°C	17°C	19°C	12°C
8	8°C	4°C	28°C	14°C	27°C	19°C	29°C	18°C	24°C	14°C
9	16°C	3°C	28°C	16°C	27°C	18°C	29°C	19°C	27°C	13°C
10	18°C	3°C	20°C	14°C	27°C	19°C	25°C	18°C	24°C	9°C
11	23°C	10°C	26°C	16°C	28°C	21°C	25°C	18°C	24°C	7°C
12	18°C	9°C	30°C	17°C	30°C	21°C	24°C	18°C	20°C	9°C
13	21°C	7°C	31°C	17°C	33°C	22°C	24°C	16°C	17°C	10°C
14	25°C	14°C	29°C	17°C	30°C	19°C	26°C	17°C	21°C	11°C
15	19°C	6°C	25°C	17°C	31°C	22°C	26°C	17°C	24°C	14°C
16	19°C	5°C	28°C	18°C	33°C	21°C	26°C	18°C	19°C	9°C
17	25°C	9°C	21°C	15°C	31°C	21°C	23°C	16°C	23°C	8°C
18	29°C	12°C	23°C	14°C	31°C	21°C	27°C	17°C	25°C	9°C
19	29°C	14°C	25°C	14°C	32°C	21°C	29°C	17°C	26°C	14°C
20	27°C	15°C	23°C	13°C	30°C	18°C	28°C	16°C	19°C	12°C

21	28°C	14°C	23°C	13°C	31°C	20°C	22°C	18°C	24°C	12°C
22	32°C	17°C	23°C	13°C	32°C	20°C	27°C	17°C	18°C	12°C
23	19°C	7°C	25°C	15°C	32°C	22°C	27°C	17°C	17°C	8°C
24	18°C	8°C	26°C	15°C	33°C	19°C	29°C	18°C	21°C	10°C
25	19°C	10°C	27°C	11°C	32°C	21°C	26°C	18°C	18°C	12°C
26	12°C	6°C	24°C	17°C	32°C	21°C	26°C	16°C	23°C	13°C
27	22°C	9°C	26°C	16°C	33°C	22°C	26°C	15°C	21°C	12°C
28	13°C	9°C	25°C	15°C	33°C	22°C	25°C	15°C	23°C	11°C
29	18°C	9°C	27°C	15°C	32°C	22°C	24°C	12°C	23°C	3°C
30	22°C	9°C	27°C	17°C	29°C	21°C	26°C	11°C	18°C	5°C
31	25°C	14°C			33°C	23°C	25°C	11°C		

Table S2. Daily Temperature Chart (May-September 2022).

	**May**		**June**		**July**		**August**		**September**	
	**Daily Maximum Temperature**	**Daily Minimum Temperature**	**Daily Maximum Temperature**	**Daily Minimum Temperature**	**Daily Maximum Temperature**	**Daily Minimum Temperature**	**Daily Maximum Temperature**	**Daily Minimum Temperature**	**Daily Maximum Temperature**	**Daily Minimum Temperature**
1	12°C	0°C	21°C	8°C	25°C	21°C	32°C	21°C	20°C	6°C
2	16°C	6°C	35°C	23°C	28°C	20°C	33°C	22°C	25°C	10°C
3	25°C	10°C	33°C	23°C	29°C	20°C	31°C	21°C	27°C	12°C
4	28°C	11°C	19°C	15°C	29°C	18°C	30°C	23°C	17°C	10°C
5	27°C	9°C	23°C	12°C	30°C	22°C	29°C	19°C	20°C	11°C
6	18°C	3°C	21°C	10°C	30°C	21°C	31°C	20°C	22°C	9°C

7	14°C	3°C	18°C	11°C	24°C	20°C	25°C	19°C	24°C	11°C
8	21°C	1°C	19°C	8°C	30°C	21°C	26°C	13°C	28°C	11°C
9	23°C	5°C	22°C	12°C	29°C	19°C	27°C	16°C	28°C	14°C
10	25°C	10°C	23°C	12°C	29°C	18°C	29°C	18°C	29°C	15°C
11	16°C	5°C	25°C	15°C	29°C	15°C	24°C	16°C	28°C	14°C
12	16°C	3°C	26°C	16°C	28°C	17°C	27°C	16°C	26°C	12°C
13	19°C	3°C	25°C	16°C	28°C	20°C	27°C	15°C	26°C	13°C
14	11°C	4°C	24°C	11°C	37°C	27°C	24°C	18°C	22°C	14°C
15	20°C	6°C	23°C	16°C	25°C	16°C	22°C	17°C	17°C	13°C
16	20°C	5°C	25°C	17°C	24°C	17°C	26°C	14°C	18°C	15°C
17	24°C	8°C	24°C	17°C	26°C	18°C	28°C	16°C	22°C	12°C
18	24°C	9°C	24°C	16°C	26°C	18°C	28°C	19°C	22°C	6°C
19	23°C	10°C	27°C	17°C	25°C	16°C	22°C	13°C	18°C	2°C
20	26°C	9°C	29°C	19°C	26°C	17°C	25°C	12°C	17°C	3°C
21	27°C	11°C	25°C	17°C	26°C	19°C	28°C	17°C	22°C	8°C
22	25°C	7°C	25°C	17°C	29°C	18°C	17°C	11°C	21°C	12°C
23	28°C	16°C	24°C	19°C	28°C	19°C	23°C	10°C	10°C	6°C
24	27°C	13°C	24°C	18°C	25°C	18°C	24°C	11°C	20°C	7°C
25	16°C	7°C	26°C	15°C	29°C	20°C	24°C	11°C	23°C	8°C
26	15°C	6°C	28°C	17°C	32°C	20°C	20°C	8°C	25°C	7°C
27	19°C	7°C	26°C	23°C	31°C	20°C	21°C	7°C	26°C	9°C
28	29°C	12°C	25°C	19°C	32°C	22°C	23°C	13°C	27°C	6°C
29	27°C	11°C	35°C	21°C	29°C	21°C	19°C	13°C	26°C	10°C
30	20°C	11°C	27°C	19°C	30°C	21°C	23°C	8°C	26°C	12°C
31	21°C	11°C			27°C	18°C	26°C	11°C		

Table S3. Plant traits under different experimental treatment conditions.

		CK	Z	H	W
2022/7/17	TC (g kg^{-1})	432.76 ± 16.90	473.67 ± 21.38	495.92 ± 16.01	488.63 ± 17.61
2022/9/4	TC (g kg^{-1})	489.74 ± 14.23	534.81 ± 10.41	554.96 ± 19.70	581.88 ± 33.48
2022/7/17	TN (g kg^{-1})	12.39 ± 1.43	11.78 ± 1.38	14.23 ± 1.32	17.47 ± 2.16
2022/9/4	TN (g kg^{-1})	11.52 ± 0.14	12.87 ± 0.43	15.36 ± 0.59	16.93 ± 0.07
2022/7/17	TP (g kg^{-1})	1.32 ± 0.06	1.56 ± 0.10	1.93 ± 0.16	2.07 ± 0.25
2022/9/4	TP (g kg^{-1})	1.45 ± 0.11	1.9 ± 0.25	2.22 ± 0.22	2.63 ± 0.08
2022/9/4	species richness	2 ± 0.47	4 ± 0.47	5 ± 0.47	6 ± 0.82
2022/9/4	Shannon–Wiener index	0.24 ± 0.06	0.66 ± 0.07	1.28 ± 0.15	1.51 ± 0.09
2022/9/4	biomass (g m^{-2})	1145.33 ± 81.99	1250.67 ± 120.86	1605.33 ± 91.53	1716.44 ± 136.10

Table S4. Soil properties under different experimental treatment conditions.

		CK	Z	H	W
2022/7/17	SOC (g kg^{-1})	589.59 ± 33.37	579.19 ± 21.62	665.98 ± 20.48	788.49 ± 19.94
2022/9/4	SOC (g kg^{-1})	576.70 ± 47.16	586.69 ± 27.85	674.04 ± 26.80	806.17 ± 55.87
2022/7/17	TN (g kg^{-1})	14.98 ± 0.36	15.02 ± 0.31	16.03 ± 1.03	15.37 ± 1.08
2022/9/4	TN (g kg^{-1})	13.75 ± 2.43	14.12 ± 0.97	15.04 ± 2.08	15.27 ± 1.15
2022/7/17	TP (g kg^{-1})	1.75 ± 0.09	1.76 ± 0.13	2.07 ± 0.14	2.15 ± 0.11
2022/9/4	TP (g kg^{-1})	1.81 ± 0.15	1.83 ± 0.22	2.14 ± 0.08	2.22 ± 0.19
2022/7/17	NH_4^+-N (mg kg^{-1})	59.05 ± 3.05	110.27 ± 4.00	194.52 ± 4.97	271.01 ± 3.07
2022/9/4	NH_4^+-N (mg kg^{-1})	66.94 ± 7.20	127.28 ± 7.58	215.86 ± 4.96	301.13 ± 3.25
2022/7/17	NO_3^--N (mg kg^{-1})	9.67 ± 0.30	17.71 ± 0.65	38.89 ± 0.51	66.35 ± 0.54
2022/9/4	NO_3^--N (mg kg^{-1})	10.49 ± 0.42	16.69 ± 1.79	60.28 ± 2.42	92.17 ± 0.56
2022/7/17	AP (mg kg^{-1})	5.94 ± 0.11	6.03 ± 0.30	6.82 ± 0.29	8.59 ± 0.26
2022/9/4	AP (mg kg^{-1})	5.8 ± 0.11	6.23 ± 0.64	7.5 ± 0.16	9.72 ± 0.57
2022/7/17	EC	84.4 ± 4.96	137.2 ± 4.71	183 ± 9.92	332.4 ± 3.93
2022/9/4	EC	90.2 ± 12.32	173.2 ± 12.18	232.1 ± 20.63	398.6 ± 2.74
2022/7/17	pH	5.58 ± 0.09	5.62 ± 0.12	5.33 ± 0.17	5.45 ± 0.12
2022/9/4	pH	5.64 ± 0.11	5.37 ± 0.12	5.39 ± 0.02	5.38 ± 0.15
